# Vulnerability as practice in diagnosing multiple conditions

**DOI:** 10.1136/medhum-2017-011433

**Published:** 2018-06-27

**Authors:** Lindsay-Ann Coyle, Sarah Atkinson

**Affiliations:** Institute for Medical Humanities, Durham University, Durham, UK

**Keywords:** medical humanities, primary care, social science, patient narratives

## Abstract

The paper contributes to contemporary understandings of vulnerability by expanding their scope with an understanding of vulnerability as generated through institutionalised practices. The argument draws on experiential accounts of navigating the practices of diagnosis by people living with multiple conditions of ill-health and disability. Vulnerability as a concept is used widely across different domains and conveys a multitude of meanings. Contemporary biomedicine, and its associated health systems and services, understands vulnerability mostly as inherent to particular physical and mental bodily conditions that put people at risk of ill-health or emotionally fragility. This may combine with a more epidemiological understanding of vulnerability as the experience of certain population groups subject to entrenched structural inequalities. Philosophers and feminists have argued that vulnerability is a universal experience of being human while political commentators have explored its potential as a resource for resistance and action. Diagnosis within medicine and psychiatry has been the subject of extensive social analysis, critique and activism. The paper draws on first-hand experiential accounts collected through face-to-face interviews with people living with multiple conditions about their experiences of diagnosis, mostly at the primary care level. We identify five aspects to diagnostic practice that are harmful and exacerbate the experience of vulnerability: temporal sequencing; diagnostic authority; medical specialisation; strategic symptom selection; medical isolation. However, these diagnostic practices are not best understood only in terms of the power asymmetries inherent to the medical consultation, but are embedded into the very institution of diagnosis. The paper thus proposes a combined approach to vulnerability that recognises it as a universal condition of humanity but one that becomes animated or amplified for some bodies, through their own inherent incapacities or the external structures of inequality, and through the practices of medicine as situated in particular times and places.

## Introduction

Vulnerability has become a widely used concept across many disciplines and fields of study. The various entry points to thinking about vulnerability, its uptake across a range of policy contexts and its potential as a label for specified individuals, groups, places or experiences, make it a heavily freighted term carrying multiple layers of meanings, implication and policy responses. Despite diversity in how the concept is mobilised, vulnerability is understood to reference a risk of or susceptibility to harm and it may also reference a limited capacity or resilience to absorb, adapt to or recover from that harm.^1^ In research and practice related to care, the growth in the presence and influence of the concept within policy, practice and identity is argued to constitute a ‘vulnerability zeitgeist’.^2^ This prominence, or ‘intellectual fashion’,^2^ indicates a concomitant need for a critical engagement with both the concept and its applications.

This paper seeks to interrogate contemporary understandings of vulnerability through dialogue with empirical first-hand accounts of the experiences of diagnosis at the primary care level for those living with multiple conditions of ill-health and/or disability. Four understandings have relevance in the context of social care and welfare: vulnerability as embodied difference; vulnerability as entrenched inequality; vulnerability as universal and vulnerability as resource for resistance. The primary empirical data indicate that vulnerability is constituted by inherent physical and mental bodily characteristics, from external structures of inequality and through medicine’s own institutional practices of diagnosis in the face of the challenges presented by the ambiguities and multiplicities of those living with a diverse range of symptoms. We are using the term practice here to capture the established codes, formal and informal, through which medicine is enacted and delivered. As such, the paper draws attention to the concrete specificities that render some bodies more vulnerable than others and how these play out through the practices of diagnosis.

The next section reviews the four approaches to vulnerability, starting with the dominant use within contemporary medicine and its systems, services and policies. This is followed by a description of the research design, an introduction to the medical case study population, those living with multiple conditions and an overview of research on diagnosis as a social phenomenon. The empirical material is presented and discussed through three examples of challenges faced by patients with multiple conditions in negotiating and managing multiple diagnoses, mostly at the primary care level. A further section explores the specific argument that vulnerability may constitute a resource for resistance. The paper closes with a wider discussion and concluding reflections.

## Vulnerability

In medicine, as in much of social care and welfare, vulnerability is typically used in relation to particular groups and individuals. These categories of vulnerability define those in need of special protection and different treatment from the wider population. This imaginary of vulnerability as a state of exception is underpinned by the contemporary liberal and neoliberal framings of the desirable person as autonomous and independent, exercising responsibility for their own and their dependents’ health and health needs.^3^ In turn, this imaginary underpins the definition of a social care system that functions as an enclave, a safe space, through which people are relieved, both temporarily and permanently, from the routines and responsibilities of everyday social living.^4^ Those seen as vulnerable include people living with certain categories of disease and disability for which they require additional support, and those living in circumstances of social, economic and environmental deprivation. Moreover, the term ‘vulnerable’ has been extended in its applications since the early 2000s to encompass a far wider set of social and experiential conditions, with a concomitant shift in attribution from characteristics to individuals at risk, and often with an emphasis on psychoemotional intervention.^5 6^ Labelling a range of bodily states, social and demographic categories, geographical settings and so forth as vulnerable has at least two important potential consequences. First, when vulnerability is ascribed as a special status, it is located as a property of those bodies in physical and mental states seen as diverging from an expected and desirable norm of autonomy, responsibility and accountability. While this categorisation of vulnerability can facilitate access to care for significant needs, it may also bring attendant challenges in terms of agency, human rights, research ethics and social justice.^7^ Bodies defined as not fitting a norm may be easily stigmatised if, through being granted the status of exception through a lens of vulnerability, they appear to fall short of the autonomy and agency expected of full citizens.^8^ Moreover, not meeting bodily, social or functional norms is often closely connected to experiencing shame which can have far-reaching consequences for generating and managing ill-health or disability.^9–12^ Second, a shift in attention from the processes of inequality that structure material deprivation and social exclusion to a condition of vulnerability that is individualised and experiential drives a prevention agenda that is operationalised through individually targeted strategies for resilience and psychological health and that is evident in a rapidly growing industry of well-being.^8 13 14^

The dominant valorisation of an autonomous and independent self-actualising subject, exercising competence and capacity over their own lives, arguably presents a diminished vision of humanity.^15 16^ By contrast, those championing a social model of disability present as a central argument that it is environments, physical and social, that disable and render people vulnerable, not the bodily differences in themselves, bringing attendant implications for policy in relation to buildings, access and facilitating action. This focus on external environments treats vulnerability as situational, in which it is the structural circumstances that render individuals, populations and places vulnerable, not the characteristics of individual bodily variabilities.^1^ This is a variant on social and political approaches in which it is entrenched structural inequalities that render individuals, population groups or global regions vulnerable. In health research, this approach is captured through the social determinants of health.^17 18^

A different engagement with a concept of vulnerability comes from philosophy and the feminist ethics of care. These share the dominant understanding of vulnerability as an existential state of being. However, instead of treating this state of vulnerability as undesirable, pathological and a failure of citizenship, vulnerability is seen as a universally shared characteristic of being human, and as inevitable, desirable and beneficial.^3 19 20^ Human beings are relatively weak physically and, thus, are inherently vulnerable to others through an essential dependency on collective and social living to survive and thrive. When vulnerability is recognised as the essential existential state of being human, then policy should focus on enabling caring relations and practices as its primary goal, thereby valorising relational and emotional skills such as empathy, support, care and sharing in building cohesive and more equal societies.^19^ These various positions are not, of necessity, oppositional. Although all humans may be inherently vulnerable, its expression may be what Fineman terms episodic and shifting, for example, across the life-course.^16^ Kittay^21^ argues that although vulnerability is part of being human, and not an exceptional condition, people are not what she terms ‘symmetrically situated’ because settings, relations and capacities are deeply unequal. Attention is thus drawn to the external conditions that structure entrenched inequalities and emergent precarity within an agenda for social justice and progressive social policy. Finally, Levinas takes this argument for universal vulnerability further and brings it back to autonomy by positioning it as a condition that is prior to autonomy, as the very process on which a variant of autonomy depends.^22^ This generative role for vulnerability in cultural and political expression is echoed in the recent work by Butler *et al* on the generative role for vulnerability in the agency of resistance.^23^

The vulnerability of patients within the medical consultation has been explored and explained in relation to a combination of the inherent bodily experience and the inequalities of power between physician and patient given the physician’s specialist knowledge and gatekeeping role, through diagnosis, to legitimate illness and its treatment.^24^ While this makes some acknowledgement of how structural inequalities engender vulnerability, this is within the particular and small-scale context of professional expertise, a context in which differential bodily experiences are rendered vulnerable through being explicitly pathological. A broader socially and politically inflected analysis interrogates the ways in which particular times, settings and practices may interact with particular bodily states and thereby expose and reinforce inherent vulnerability and render those bodies at particular disadvantage. This approach is explicitly alternative to that in which the vulnerability inheres to given categories of bodies or specific settings, usually through some variant of impaired autonomy, and instead offers a relational analysis in which the complexities of different bodily states become more or less vulnerable always and necessarily through interaction with the concrete specificities of time, place and relations.^1^

We examine how these concrete specificities that render some bodies more vulnerable than others play out through the processes of clinical diagnosis. We document a set of experiences in relation to diagnostic encounters of those living with multiple conditions. Those living with multiple conditions present particularly complex bodily states and the study documents how, operating within a medical approach of the exceptional body, the conditions are variously simplified, recategorised, managed badly or dismissed. We argue that, instead of conceptualising vulnerability in terms of the incapacities of bodies, it is rather the institutional processes and practices of diagnosis that disclose an incapacity to manage these complex bodily states and themselves constitute the experiences of vulnerability expressed by the research participants.

## Methodology

### Research design and ethics

The field of medical humanities has generated a rich tradition of attention given to the medical consultation and the diagnostic moment.^25^ However, the focus has been interpersonal and particularly on the physician as communicator and the possibilities for empathy.^26 27^ There has been far less engagement with how the different participants in the consultation are themselves embedded within wider social structures^28^ and almost nothing on the institutionalised dimensions of diagnostic practice as opposed to physician competence. One of the major contributions, however, by the medical humanities to understanding the medical encounter and living with illness, has been an appreciation of different forms of knowledge and evidence, and in particular, the value, complexities and constraints of evidence from first-hand experiential accounts.^28 29^ In a review of the range of contributions of the medical humanities, Whitehead and Woods foreground experience, and especially the illness experience, as one of three ‘Es’, alongside ethics and education, that have characterised and shaped the emergence of the field.^27^ While others in the social sciences and humanities also privilege experiential knowledge, medical humanities brings to such accounts a focus through interpretation, interrogation or both, on those concepts that are central to contemporary medical practice and policy.

The focus of the research on multiple conditions developed out of an initial interest with examining the apparent boundaries between mental ill-health, physical ill-health, chronic ill-health and disability.^30^ The focus in this paper on vulnerability emerged from a wider attention to the experiences of living with multiple conditions and so we have only accessed experience of diagnosis from one side of the encounter, the perspective of the patient. There are clearly many cases of excellent practice in managing those presenting with multiple conditions and complex reasons for the decisions physicians make in the consultation which we have not accessed in this study.^31 32^ Accessing those who are living with any of these conditions on a chronic basis can be challenging and we started out by working with a mental health resource centre in the North-East of England, The Waddington Street Centre, in Durham City. Users included those across all ages and social and economic classes of the region. The area around Durham has a relatively small Black, Asian or Minority Ethnic population beyond the University and the centre users were predominantly white. People attending this centre come from the local area, with the centre located only 5 min walk from the train and bus stations, and are typically referred there by a community psychiatric nurse, social worker or other health and social care professional. As a result, all research participants recruited through the centre include at least one form of mental ill-health among their experiences of multiple conditions. This is highly significant for research on multiple conditions as there is a well-documented connection between mental ill-health and physical health outcomes including excessive premature mortality.^33^ A further characteristic of the sample is that it is possible that those referred to The Waddington Street Centre are those particularly dissatisfied with conventional medical care and, as such, may offer a critical voice. The Waddington Street Centre provides life skills and opportunities aimed at either maintaining or improving a person’s mental health. This includes offering courses (such as art, poetry, music, cooking and sports), as well as providing emotional support. One of us, Lindsay-Ann Coyle, volunteered regularly at the centre, building everyday relationships with the users and building awareness and sensitivity towards the range of issues the centre users faced. Lindsay-Ann worked as a volunteer at the centre’s café one morning a week. This involved making tea and coffee and serving snacks, all the while chatting to the many service users and staff who use the space. Getting to know people helped to build trust, as a member of staff explained:

… by giving us extra time as a volunteer on top of your role as a researcher and—you know—people really do value that sort of thing because it makes them believe that you actually want to be here rather than—without sounding too dramatic—rather than you’re using them to get what you want… I think people feel that you’ve invested something of yourself into the organisation as well.

Although becoming a volunteer did help to increase recruitment to the project, it had many other benefits. Alongside helping to build trust, it also created the opportunity to informally discuss the progress of the research with people visiting The Waddington Street Centre’s café, indeed, the project became a common talking point among the café users. Given Lindsay-Ann’s positionality as a female researcher, we might have expected that female participants would feel more comfortable being interviewed than the men and be more open about their experiences, but there was no evidence of this. Indeed, our male respondents were equally open regarding the personal and intimate challenges of their multiple conditions.

Potential research participants at The Waddington Street Centre were recruited through posters, conversations and word-of-mouth. The research started by seeking one-to-one interviews with users who identified as negotiating anxieties, such as obsessive compulsive disorder (OCD), panic disorders and post-traumatic stress disorder (PTSD). The project’s focus on multiple conditions emerged during these first interviews as participants repeatedly described how they were living with the effects of a number of other symptoms and conditions, including additional mental health problems and pain, autoimmune diseases and many others. Therefore, although the starting point for recruiting participants was through the lens of ‘anxieties’, the research developed to consider people’s experiences of living with multiple conditions of illnesses and/or disability. For this, we recruited 12 participants through the centre; three further participants who did not attend the centre were recruited by a snowball sample through researcher and participant contacts and agreed to participate in the research and discuss their experiences. Participants’ age ranged between 18 and 70 years and included slightly more women than men. Most studies would provide a breakdown of the characteristics of people who participated in the research, such as age, gender and ethnicity of participants. However, because the name of The Waddington Street Centre is used in this research, this information will not be released in order to protect the anonymity of participants as far as possible. This issue is discussed later in relation to ethics.

Data were generated through semi-structured interviews, working with a list of themes to prompt the discussion. This method offers the potential to take a careful and compassionate approach to the conduct of research, in which people feel comfortable narrating their life experiences.^34^ In practice, each participant was asked about given topics, such as ‘mobility’, ‘healthcare’ and ‘home’, with a view to encouraging very broad discussion around these themes. This approach proved an excellent entry-point to the discussions as it provided some structure to the interview and enabled participants to narrate detailed personal accounts of their experiences and introduce additional topics. Here, the position and interpersonal exchanges of researcher and participant have to be negotiated and we understand these in terms of power,^35^ belonging^36^ and anxiety.^30^ Our participants had prior connotations of an interview and of the use of recording devices^37^ in relation to accessing work and services. Lindsay-Ann’s volunteer work in the centre cafe, her relatively junior status as a young woman and her own quiet manner, emphases on commonalities and processes of consent aimed to mitigate the associations as far as possible. Nonetheless, past associations expressed themselves explicitly when interviewing Jack:

When I met him in the corridor a few minutes later, he asked if he had done alright and if he had passed (as if was an exam). I explained that it wasn’t an exam but that what he had said was very helpful to me and the project. I thanked him again for his participation.’

(From Lindsay-Ann’s research diary)

Building commonalities with participants in part depends on the extent of being an insider or outsider from the participants’ perspective. While Lindsay-Ann worked at the centre and had some insider status as a volunteer, she nonetheless was not referred to the service facilities. This was expressed explicitly by one of the participants, Michael:

… you sit round a table with people who have the disability and I’ll talk about it perfectly happily about it as a disability. But when it’s in front of people who are not disabled you tend to hold back unless you’re being asked questions (laughs and points to Lindsay-Ann) because it is—it’s the embarrassment sometimes. Em and guilt—again… keeps coming back…

The relations of power and belonging are infused in this study with feelings of anxiety, which many participants experienced as part of their conditions regardless of the extra anxieties of research participation. Angela expresses this in commenting on her interview in terms of both negative and positive outcomes:

…oh my goodness I know I’m going to analyse this conversation for the rest of my life (laughs)—em—and say I was too open, I was too forward, I was- talked too much. Because I’m not usually this energetic and I don’t usually talk this much and it’s kind of thrown me a bit—em… it’s an achievement. I’ve been out, I’ve did it, I’ve survived another day. But there’s the anxiety over all the things that I’ve said and done and how I’ve looked and everything like that.

These three considerations of power, belonging and anxiety are acknowledged as contributing to the context in which empirical data were produced as part of this research.

Interviews were transcribed and analysed through an expanded set of themes beyond those used to prompt discussion and which emerged from participant accounts of their experiences. Analysing qualitative data necessarily involves making decisions about how to understand the voices of participants which we endeavour to undertake here with a scepticism of clear-cut categories. As social scientists, we did not come to the study with a medical background but rather viewed the range of symptoms, diagnostic labels and experiential accounts as equally valid in terms of our analysis. As such, we seek to counter pervasive—but troublesome—conceptions of bodily difference that ‘deviate’ from the norm and allow for the contradictions, ambiguities, interdependencies and uncertainties that shape understandings of illness and disability.

In presenting the analysis for this paper, we chose to draw on a small number of individual stories through extended extracts rather than many short comments. Those interview extracts selected reflect experiences that many of the participants viewed as difficult and illustrate such difficulties particularly well. By chance, all the extracts selected were from female participants which may reflect a greater sensibility to interpersonal relations. However, the wider analysis did not reveal noticeable differences by gender in the kinds of experiences specified as difficult. The study faced a particular ethical dilemma in relation to anonymity.^38^ The collaborating organisation for the research, The Waddington Street Centre, required that their name would be used in publication as a condition of entry to the site. Thus, while we could endeavour to maintain participant anonymity as much as possible, we also made clear that the name of the centre would be used in any outputs produced and could lead to individual participants being identifiable. In the context of this research, maximising anonymity involves weighing up competing needs. Given the research focuses on highly personal individual experiences, we are very cautious about what information we reveal about the participants. First, we do not reveal the particular combination of illnesses and/or disabilities with which any given participant is living with the exception of the relatively frequent combination of chronic pain, anxiety and depression. Second, we also reveal very few other identifying characteristics, such as age and race. This does limit the depth of insight that comes from structural characteristics of identity, which will remain a challenge for any research on the experiences of people with multiple conditions.

The research design, recruitment, process and data management were approved through the procedures for ethical scrutiny of our home university, which are fully compliant with the Research Councils UK guidelines. In reporting specific examples from the set of interviews, all names have been changed in line with standard social science practice. We have analysed the interviews as individual cases, rather than looking for cross-cutting themes across the set. Each participant offers a distinct set of experiences, although common experiences, challenges and pathways forward do emerge.

### Multiple conditions and diagnosi*s*

The number of people living with multiple forms of ill-health and disability is rapidly increasing; in the UK alone, this number is predicted to increase over one decade, from 1.9 million in 2008 up to 2.9 million by 2018.^39 40^ Understanding and managing multiple experiences of ill-health and disability constitute a major emerging challenge to how contemporary medicine is practised. There is a large and growing literature emerging on the challenges of managing multiple conditions from the perspective of physicians and related health professionals, but there has been almost no exploration of what it means to actually live with multiple conditions based on the first-hand accounts of such experiences. In the UK, growing awareness of the challenges for medical practice have prompted a review and publication of new clinical guidance.^41 42^ The guidance specifically addresses challenges for the medical practitioners including the management of the physician’s consultation times when confronted with multiple conditions and the dangers of multiple discrete prescriptions for a suite of different diagnoses. At the same time, finding a language to describe this experience is challenging, given the sociological arguments for differentiating disease, ill-health and disability. Here, we have avoided the overly medicalised term of ‘multiple morbidity’, and the overly clumsy phrasing of ‘multiple ill-health and/or disability’ and have settled on ‘multiple conditions’ as a practical compromise.

A social approach to analysing diagnostic practice was first specified by Blaxter in 1978 who argued it constituted both an event and a process.^43^ At its core, the practice of diagnosis demands judgement by medical professionals about the relevance and importance of particular symptoms which can be packaged together to move to naming an illness category and indicating a set of treatment options. This exercise of clinical judgement is a key to how medicine ‘legitimates an ill body by naming a specific disease process and so permits access to particular modes of treatment’.^44^ The considerable body of socially informed research on diagnosis has explored the impacts and management of the diagnostic moment,^45^ the various roles of diagnosis as legitimation, stigma and affliction,^46^ the clues and cues in the consultation that anticipate the delivery of a diagnosis^47^ and the ways in which diagnosis is a negotiated process between patient and practitioner.^48^ A relatively empowering process of negotiation becomes more problematic where the performance or discourse of the patient has greater influence on the diagnostic outcome than any objective diagnostic criteria.^49^ Activist and peer-support groups have challenged expert diagnostic systems where certain conditions are not recognised at all and where the boundaries and labels of diagnostic categories are contested, changed or rejected.^50–53^ Questioning the validity of diagnostic categories has been particularly associated with, but by no means limited to, mental ill-health conditions, most recently in the wake of the Diagnostic and Statistical Manual of Mental Disorders, Fifth Edition manual.^54–57^ Manuals and coding protocols may also be used variably across different settings as to whether diagnostic procedures, categories and treatments are followed strictly or used more as helpful guidance towards treatment.^58 59^ Analysts of diagnostic procedures within mental healthcare have called for greater allowance for ambivalence and permeability of categories,^60 61^ a call that may be instructive for diagnostic practice more widely. Nonetheless, despite this history of challenge to diagnosis within mental health, our participants’ encounters at the primary care level appeared to a large degree to seek and to hold categorical diagnostic labels suggesting the enduring institutionalisation of diagnosis for accessing care.

This body of research on the sociology of diagnosis has thus generated sensitive and critical insights in understanding contemporary medical practice; however, the work is predominantly on specific conditions or health programmes. We know very little about the experiences and impacts of diagnosis for those negotiating multiple symptoms, the multiple effects of shame and stigma^62^ and the multiple potential categories for who the various events and processes of diagnosis are continuously re-enacted and re-experienced. In the next sections presenting our results, we have drawn on a small number of individuals to illustrate some of the experiences that were discussed in relation to the diagnostic consultation. These are presented through three thematic headings of ‘hidden symptoms’, ‘competing symptoms’ and ‘conflicting conditions’.

## Results

### Hidden symptoms

Diagnosis, as a practice of matching sets of symptoms to categories of ill-health or disability, becomes complicated when particular symptoms may be variably present or hidden in the bodies of those negotiating multiple conditions. The presence of multiple conditions sometimes obscures particular presentations of illnesses and disabilities at particular times and in particular spaces. As such, attending to how those living with multiple conditions negotiate their medical encounters reveals fluctuations in which particular illness or disability is prominent relative to other illness and disability.

Our participants’ accounts reveal how they are vulnerable with respect to their capacity to access appropriate care through a diagnosis, and how the biomedical model of diagnosis itself may be understood as vulnerable with respect to dealing with multiple conditions in the impossibility of isolating the presentation of any one illness or disability during the clinical encounter. The emphasis here is on the health system, not the specific physicians who practice within its framework. This notion of a vulnerability as an inherent incapacity of both patient and system in the face of multiple conditions is illustrated by Kirsty’s account of her experiences of OCD, anxiety and depression:

… the depression was the first thing that was obvious when I was really ill. I just wasn’t functioning and then, as the anti-depressants kicked in, … I was beginning to do more things. And it then became more obvious that it wasn’t the [depression]. Say I was making a cup of tea—the depression was really bad, I couldn’t think clearly. It was like being in the dark, in the fog and just not being able to think clearly. So I couldn’t think to do things in the right order—like fill up the kettle then get the teabag. (I was) just doing things in the wrong order and not being able to think and not being able to coordinate. And as that began to be slightly more automatic and a bit clearer, it became obvious that I was then having to get the kettle in the right place and the mug and do something with the teabag and time it and all these sort of things.

Kirsty’s experience illustrates how a diagnosis of one condition is complicated by the existence of others. Her OCD was not recognisable by either herself or other people until her depression was treated. Kirsty had difficulty making a cup of tea in both instances but she narrates a different cause to the difficulties in each case. Kirsty indicates that two conditions can be present but that one of them effectively hides the other, thereby demonstrating that one symptom or diagnosis may be more obvious, or privileged, in the consultation at any given time. In Kirsty’s account, her experience of OCD is emphasised at one point but her experience of depression is emphasised at another. This idea of ‘hidden symptoms’, or symptoms that become more or less obvious in relation to other conditions undermines a model of diagnosis that relies on the presentation of all symptoms. Moreover, Kirsty’s experience destabilises particular medicalised understandings of illness, whether chronic or acute, as a linear narrative in which the illness causes symptoms which can then be diagnosed and hopefully treated. Instead, Kirsty’s account of the everyday effects of her ill-health points to a very different and experiential understanding of time as ‘linked to performed activities and processes, resulting in an individualised temporality of change'.^63^ From a biomedical perspective, Kirsty may be vulnerable to part of her health problems being missed, but, nonetheless, her multiple conditions are addressed through the established practice of diagnosis, although through a sequential process of multiple diagnoses as the treatment of one reveals the expression of the other. Despite an apparent destabilisation of medical linearity, in the clinic at least, it is re-established to a certain extent through this sequential process of multiple diagnoses. But from Kirsty’s perspective, what is of concern is the difficulties she faces in daily life such as making a cup of tea, difficulties experienced as a whole, as one problem not as multiply layered problems that may take time to excavate.

### Competing symptoms

Sociologists of diagnosis have demonstrated how the process of diagnosis may more often than not be a process of negotiation between the physician and the person presenting with symptoms.^48^ However, negotiating what might be the nature of the experience of ill-health becomes problematic when disagreement arises between patient and practitioner about the relevance and importance of particular symptoms and treatments. For people who do not describe a coherent set of symptoms that may neatly translate into a single diagnosis, or even several diagnostic categories, the relative importance attached to particular symptoms becomes a point of contestation between patient and health professional. A number of our participants described how engaging with the process of negotiating the diagnosis involves constantly weighing up the relevance and importance of particular symptoms to be presented. But Stephanie illustrates the vulnerability of the patient voice through the structural inequalities and asymmetries between physician and patient in negotiating the diagnosis, inequalities that reflect an inevitable tension between valuing the expert’s knowledge and skills and valuing the patient’s lived experiences; when there is a difference of opinion on which symptoms the patient and the professional consider most urgent, professional opinion carries the greater weight:

So when I was diagnosed with clinical depression and suicidal tendencies at that time, I felt like the bulimia just got swept under the carpet like it didn’t matter. [But] in terms of what I actually did and what was affecting my life on the most frequent basis, it was definitely bulimia. But the one that they were worried about, in terms of severity of consequences, was depression and suicidal tendencies so I sort of felt like some of the things that I did that I felt were about the bulimia were classed as being about the depression now. And I’m not saying that they should have been pushed back into the bulimia category. But, rather, you can’t neatly define up what is agoraphobia, what’s bulimia, what’s depression, what’s OCD or anxiety disorder.

As well as recognising the inevitable power differences in a process of negotiating a diagnosis, Stephanie’s experience demonstrates a politics of urgency that is negotiated in clinical encounters. Even though Stephanie experienced bulimia, she reports that the diagnosis of depression was viewed by her doctor as the important focus; Stephanie was treated as more vulnerable to her incapacities from depression given the associated suicidal tendencies and in greater need of immediate protection from the depression than her bulimia. But Stephanie’s own perception is that bulimia is the more urgent condition as she is affected by it most frequently. Here then we see the conventional medical construction of vulnerability as bodily incapacity in relation to life-threatening conditions in which the patient is in need of protection and in which the physician is in the privileged position of knowing better. Furthermore, within this context of ceding authority over urgency, Stephanie describes a situation where the boundaries between the depression and bulimia experiences are redrawn by her physician. Experiences she attributed to bulimia were categorised as symptoms of depression through the medical encounter. Redrawing the boundaries in this way is part of the process of establishing and re-establishing hierarchies of bodily difference that directly relate to the perceived urgency of treating particular symptoms. This tension over establishing the urgency of treating any one illness or disability over another reflects the compartmentalisation of particular diagnoses and again renders vulnerable patient bodies and modes of medical practice in regard to the challenges it presents for engaging the conditions through a more holistic frame.^64^

A variation on this point is that sometimes physicians may aim to focus on particular symptoms in isolation from others. The presence of multiple symptoms that were difficult to categorise led to a situation where Vicky disagreed with the diagnosis offered to her by the health professionals:

…I sometimes refer to myself as having OCD and I sometimes refer to myself as having an eating disorder…and for me that seemed quite natural because, when I looked it up, to have an eating disorder you have to eat for psychological reasons—not for physical reasons…. well that’s me down to a T [that’s me ‘exactly’]. And I think I have an eating disorder, as well as OCD. And I had an appointment with an eating disorder clinic which is actually where I wanted to go—I wanted that over CBT. I wanted to talk to people—[about] the physical eating side of it because that was the problem, not the behaviours. Their reason for turning me away from an eating disorder clinic was because I didn’t have a problem with weight or body image and that made me quite angry that the definition of an eating disorder in the eyes of the NHS is—you can go to an eating disorder clinic if you have an issue with weight or body image. And that was something I struggled with on a day to day basis was: ’I have OCD, what about eating?'… that needs to be addressed… if OCD can relate to anything I think I should have been able to go to an eating disorder clinic to talk about my relationship with food. Like, where else is better to do that, realistically, than someone trained to talk to people with eating disorders? But I wasn’t allowed to do that.

Vicky’s account of experiencing rejection and uncertainty underscores the emotional implications of negotiating multiple, contested illness and disability identities in clinical settings. Here, Vicky perceives her OCD experiences to be foregrounded by health professionals, while her eating disorder was ignored, dismissed or, at best, downgraded as relatively unimportant. The experiences of both Vicky and Kirsty reveal structural vulnerabilities in accessing care, a vulnerability that is always present in a medical consultation in which the physician acts as the gatekeeper for treatment through diagnosis. This structural vulnerability, however, may become particularly marked where the patient has multiple and disparate sets of symptoms over which physicians and patients disagree.

### Conflicting conditions

Any treatment provided to people with multiple conditions has implications for the whole emotional and material body. This point is one which exposes the difficulty that participants in this research had in ‘fitting into’ treatment spaces. The medical focus on the treatment of a single isolated condition can be hugely problematic for some of those people experiencing multiple illnesses and/or disabilities. Angela highlights some of the difficulties associated with the treatment of one illness at the expense of another:

Interviewer: and how did you find it when you were in the hospital? What did you think of it?

Angela: it was hell. It was hell because it was constant interaction with people - there was other people constantly there so, for someone with extreme social anxiety, that was just—the way I moved, everything I said, everything, even the way I lay in bed. I used to think ’oh gosh people will think I’m this or that'. And no amount of CBT can, kind of, override that because it’s almost like a gut instinct that’s there.

While Angela was in hospital to treat one of her conditions, her experience of social anxiety was particularly acute causing her huge distress. Although Angela was not physically excluded from the treatment setting because of one of her other diagnoses, she was effectively excluded. Given that Angela’s social anxiety did not seem to be accommodated in any way, such as by providing a separate space for her away from other people, it is evident that the social space is constructed for those with a particular diagnosis to the detriment of those with other conditions. And for Angela, there was little choice about her going into hospital and this forced mobility proved to be ‘hell’ for her through increasing the exposure to the circumstances in which Angela is vulnerable to experiencing anxiety. The ways in which medical science compartmentalises the body and the ways that this translates into similarly compartmentalised medical expertise with limited integration either across specialisations or between primary and secondary care is a well-rehearsed critique.^65^ It would be easy to view the problem here as resulting from Angela’s vulnerability within the treatment structures due to her own incapacities which thus put her in need of special protection and support. However, we propose instead that we need to understand that the vulnerability inheres to the institutional procedures which themselves are revealed as incapable of engaging a range of conditions even while providing treatment for one of them.

Research participants struggled further as a result of this compartmentalisation of treatment processes as corresponding to particular problematic divisions of aspects of bodily difference. A number of accounts of those living with multiple conditions reveal how this is embodied as an integrated experience rather than as a set of discrete conditions. As such, the embodied experiences of those living with what medicine terms ‘multimorbidity’ breach and blur the boundaries and categories that are fundamental to diagnosis. Kelly describes her medical encounters:

I’ve grown so weary of trying to work out which specific symptoms might be relevant to making a diagnosis of yet another condition. For instance, at my last appointment I complained about not being able to get to sleep at night. The doctor said this ‘insomnia’ (another problem to add to the list) could be caused by any one of the ailments previously listed in my medical records. It has just become impossible to isolate any one of my existing (or new) symptoms to a particular illness category.

Kelly illustrates well how those living with multiple conditions are exposed to and incorporate the medical notion of multiple sets of symptoms and diagnostic labels, so that both patients and physicians must be seen as embedded within a culture of medical practice. In dealing with the health services, they feel they must continually rework their experiences into acceptable formats for presentation. Physicians’ specific aim to try to isolate particular diagnoses from other diagnoses enacts a form of vulnerability in which people feel excluded from some diagnoses and treatment processes. This, in turn, can be hugely damaging to emotional well-being and to a sense of identity, and, unsurprisingly, some research participants no longer engaged with mainstream medical care at all.

## Vulnerability, agency and resistance

A counterargument to the presentation of vulnerability as inhering to particular incapacities, in the case here to bodily medical incapacity, is that vulnerability itself may be generative and provoke agency and resistance.^23^ For example, support groups for those living with specific diseases are common, both locally and online and activist groups lobby for diagnosis and treatments where these are felt to be unsatisfactory. There have been notable success stories that add weight to the resistance argument with relevance to our participants: the social model of disability provided a radical challenge to the notion of dependency and special category vulnerability as inherent to certain differently abled bodies^66^; mental health activists have campaigned with some success for greater voice in defining their own needs, for better access to appropriate care including service user-led services and research.^64 67–69^ But for our research participants, the potential for vulnerability in the face of diagnostic practices to generate collective action appeared limited. And the limitations came exactly from the experience of multiplicity.

On the one hand, there has been the neglect of attention to multiple conditions in both academia and biomedicine, and on the other, there is the challenge that the experiences of living with multiple conditions presents to biomedicine’s dominant conception of bodies as stable, definable and solvable. The combination of this neglect and this challenge results in isolation for those living with multiple conditions, such that, ‘The manifestations and implications of this sense of isolation cannot be underestimated*’*.^30^ Feminist approaches to research highlight the importance of attending to the ‘secrets and silences’ in the research process as well as what is said.^70^ Silences relate to specific instances, as illustrated by Angela in her interview who said, "I feel OK talking about this, but there’s other stuff that (pauses) I find shameful, but I shouldn’t……” and trails off. A deafening silence that was thus evident in the research was the absence of any sense of belonging by participants towards a broader group or collective identity, despite our interviewing people through a mental health resource centre, in whose space such identity might have been expected to emerge. The only hint of some sense of collective identity was expressed in relation to a general, broad notion of illness and disability and, now and again, in relation to an individual disease category. These included statements such as, "I have had mental health problems for many years" ‘being ill is not easy’ and specific conditions such as, "I’ve got anxiety but that is related to my IBS irritable bowel syndrome" or "I know other people with schizophrenia as well". The absence was how nobody seemed to narrate their experiences specifically in terms of belonging to a group of people living with multiple conditions. Even when participants talked about their multiple diagnoses within the interview, these were presented as unique, complex and peculiar, sometimes as awkward, upsetting or humorous, but always as an individualised set of conditions.

Perhaps more importantly, participants were silent about any sense of belonging to a broader group of people living with multiple conditions, and some were even cautious about disclosing that they had several illnesses and disabilities at the same time. Discussing what can be seen as ‘too many’ complaints was risky for participants because their credibility may be called into question. This was evident in the interview with Stephanie in which Lindsay-Ann was just going through her various ailments and Stephanie interrupted her and cut the list short with a ‘and all of the others’. The participants appeared to find making the decision about when it is appropriate to list a multitude of conditions to be fraught with difficulty, involving a careful weighing up of how other people might respond in different given contexts. This absence of a sense of belonging among the participants, becomes a problem from the deep sense of isolation associated with being the only person with such a multiply ill and disabled body, the only person who has got a range of awkward and contradicting problems and the only person whose problems are so ‘weird’ and ‘peculiar’ that they cannot be ‘solved’.

The isolation reflects one more practice of vulnerability. The medical profession recognises the challenges facing their structures and practices by multiple conditions as evident by opinion pieces in professional journals such as the *British Medical Journal* and by the recent guidelines.^36^ Nonetheless, health professionals also deal with the diagnostic and treatment challenges on an individual case basis. In the case of the physician, this is probably quite rightly so in light of the long tradition of exhortation from the social sciences and humanities for medicine to see the patient as a whole person.^71^ But the ramifications of a personalised consultation for multiple conditions within existing diagnostic practices is that the challenges multiple conditions present continue to be seen as a diagnostic vulnerability located within an individual body and as a particular and exceptional circumstance. Moreover, this positioning of diagnostic vulnerability is enacted by both the physician and the patient.

## Diagnostic vulnerabilities

Critical social scientists have elaborated how a dominant conceptualisation of vulnerability constructs a passive body in need of exceptional care and protection owing to incapacity and lack of agency.^72^ In the context of a political culture that is increasingly characterised by the individualisation of responsibility, agency and self-care,^73^ being vulnerable becomes an individualised incapacity for self-care, and more importantly, a social deficiency with respect to citizenship.^74^

In this study we find that, on the one hand, the medical system of practice enacts and enhances this individualised vulnerability but, on the other, also discloses its own incapacities to cope with the categorical ambiguities and multiplicities of those presenting with multiple conditions. Diagnosis is often seriously misrepresented as a process of simple mapping of symptoms to disease, and both social and medical studies have demonstrated how diagnostic protocols often serve more as guides than absolute frameworks. Social studies further elaborate how diagnosis is very often a negotiated outcome of the processes between physician and patient in the consultation.^48^ Nonetheless, the participants in our study reveal at least five aspects of diagnostic practice that can impede access to adequate care for their complex bodily experiences and, as such, may be seen as structuring forms of vulnerability. First, the ways in which one set of symptoms hide other symptoms structures a distinct temporality to diagnosis in which sequential diagnoses only gradually enable sequential access to different categories of care. Second, physicians hold the authority over how symptoms are grouped into diagnostic categories and which of these symptoms and categories are accorded prioritisation over others. Medical practice thus structures a diagnostic politics of urgency in relation to which associated categories of care are to be delivered. Third, the organisation of medical practice through medical specialisations structures compartmentalised diagnostic categories that inform differentiated treatment pathways, which may be unable to accommodate unrelated additional needs arising from different conditions or integrate information on care needs between primary and hospital levels. Fourth, those living with multiple conditions describe their awareness of the need for categorical strategies in presenting and performing their symptoms as part of negotiating an outcome. Finally, those living with multiple conditions often see their particular constellation of symptoms as unique, thereby structuring an experience of medical isolation, with associated constraints for collective identity and agency. While those living with multiple conditions describe and detail these practices that constitute their vulnerability in relation to accessing care, nonetheless, they internalise and share with physicians the tendency to locate their vulnerability within their problematic bodies that do not readily fit in to easily managed symptoms or classificatory systems. Moreover, the not-fitting-in of these complex bodily experiences continued to be engaged by both medical practice and those living the experiences as special, exceptional cases rather than as cases that may fundamentally undermine the entire diagnostic project. By understanding vulnerability through different lenses, alternative engagements with diagnostic challenges may be indicated.

The main alternative of viewing all of humanity as inherently vulnerable does challenge deep-rooted imaginaries of independence, autonomy and citizenship with potential impacts of reducing stigma, but it may underplay exceptional needs for care. An approach that mediates both positions focuses on how our inherent vulnerability may become animated or exacerbated under particular conditions. In our study, we have drawn attention to the importance of looking at institutionalised practices through the experiences of diagnosis by those living with multiple conditions. Diagnosis, as both event and process, is central to the practices of contemporary medicine. However, our first-hand accounts show how modes of medical thinking continue to sit inconsistently with the difficult bodily variabilities experienced by those living with multiple conditions and we suggest that this is, in part, structured through the particular way of viewing bodily difference as vulnerability. While our first-hand accounts of diagnostic practice intimate difficulties for patients from normative medical practice in terms of temporalities, boundary setting, power and gatekeeping, treatment regimes and the imposition of predetermined categories, more fundamental is the demonstration offered of the inadequacies of a concept of vulnerability that continues to position those individual bodies requiring medical and psychiatric support as having impaired capacities and in need of a paternalistic protection. In a similar mode to the lessons for diagnostic practice in medicine more widely that can be drawn from the evident shortcomings of diagnostic categories for mental health practice, attention to the complex challenges of living with and managing multiple conditions can reveal shortcomings in governing contemporary concepts underpinning medical practice. We argue that one of these concepts, vulnerability, may be helpfully reconfigured away from an understanding as being inherent to personal incapacity and impaired autonomy. In its place, we propose that existing dimensions to vulnerability be expanded by recognising the significance of institutional and normative practices. On the one hand, the medical system itself can be seen as the site of vulnerability in relation to the incapacities and structural tensions of normative institutional practice in the face of the growing challenges of multiple conditions. On the other hand, practices interact with differentiated physical and mental bodily experiences and existing structural inequalities in particular times and places in ways that realise, animate and amplify a latent universal vulnerability and that render bodies experiencing ill-health and/or disability as particularly vulnerable to harm and the inability to withstand harm.
